# Brain Age Acceleration on MRI Due to Poor Sleep: Associations, Mechanisms, and Clinical Implications

**DOI:** 10.3390/brainsci15121325

**Published:** 2025-12-12

**Authors:** Eman A. Toraih, Mohammad H. Hussein, Abdulrahman Omar A. Alali, Asseel Farhan K. Alanazi, Nasser Rakan Almjlad, Turki Helal D. Alanazi, Rawaf Awadh T. Alanazi, Manal S. Fawzy

**Affiliations:** 1 Department of Cardiovascular Perfusion, Interprofessional Research, College of Health Professions, Upstate Medical University, New York, NY 13210, USA; toraihe@upstate.edu; 2 Medical Genetics Unit, Department of Histology and Cell Biology, Suez Canal University, Ismailia 41522, Egypt; 3 Ochsner Clinic Foundation, New Orleans, LA 70121, USA; mohamed.hussein@ochsner.org; 4 Faculty of Medicine, Northern Border University, Arar 91431, Saudi Arabia; dr.a.o.alali@gmail.com (A.O.A.A.); asseel2002@gmail.com (A.F.K.A.); almglady.7e6@gmail.com (N.R.A.); turking787@gmail.com (T.H.D.A.); r8ly400@gmail.com (R.A.T.A.); 5 Center for Health Research, Northern Border University, Arar 73213, Saudi Arabia

**Keywords:** neuroinflammation, brain aging, magnetic resonance imaging, sleep–wake disorders, glymphatic system, biological clocks

## Abstract

Sleep disturbances, affecting nearly half of middle-aged adults, have emerged as modifiable determinants of brain health and dementia risk. Recent advances in machine learning applied to MRI enable the estimation of “brain age,” a biomarker that quantifies deviation from normative neural aging. This review synthesizes and critically evaluates converging evidence that poor sleep accelerates biological brain aging, identifies mechanistic pathways, and delineates translational barriers to clinical application. Across large-scale cohorts comprising more than 25,000 participants, suboptimal sleep independently predicts 1–3 years of MRI-derived brain age acceleration, even after adjusting for vascular and metabolic confounders. Objective sleep fragmentation and altered sleep-stage architecture exhibit sleep-specific neuroanatomical signatures, independent of amyloid and tau pathology, while inflammatory, vascular, and glymphatic mechanisms mediate a small fraction of the effect. Experimental sleep deprivation studies demonstrate reversibility of accelerated brain aging, highlighting opportunities for early intervention. Translation to clinical practice is constrained by methodological heterogeneity, reliance on self-reported sleep metrics, limited population diversity, and the absence of randomized intervention trials demonstrating causal reversibility. Addressing these gaps through standardized MRI-based biomarkers, longitudinal mechanistic studies, and interventional trials could establish sleep optimization as a viable neuroprotective strategy for dementia prevention.

## 1. Introduction

Structural brain aging follows predictable trajectories that can be quantified using neuroimaging biomarkers. Machine learning models trained on Magnetic Resonance Imaging (MRI) features, cortical thickness, gray matter volume, and white matter microstructure predict chronological age with mean absolute errors of 2.4–2.5 years in validation cohorts [1]. The brain age gap (BAG), defined as the difference between predicted and chronological age, indexes deviations from normative aging and captures cumulative effects of genetic, vascular, and lifestyle exposures. Positive BAG values indicate that older-appearing brains are associated with increased mortality, progression from mild cognitive impairment to dementia, and multiple neurological conditions [1,2].

Sleep disturbances affect 30–50% of middle-aged adults and are epidemiologically linked to dementia risk. The 2024 Lancet Commission identified sleep disorders as emerging modifiable risk factors, though causal mechanisms remain uncertain. Unlike immutable genetic determinants, sleep represents a potentially tractable target for intervention [3]. Recent technological advances, particularly the UK Biobank’s acquisition of over 50,000 brain MRI examinations with concurrent sleep assessments, enable a systematic investigation of whether poor sleep accelerates structural brain aging [4].

This article is a narrative review and does not follow formal systematic review or PRISMA procedures. The literature was identified through structured searches of databases such as PubMed and Embase using combinations of terms related to brain age and sleep (e.g., ‘brain age’, ‘brain age gap’, ‘sleep duration’, ‘sleep fragmentation’, ‘insomnia’, ‘obstructive sleep apnea’, ‘functional connectivity’), without restriction to a single study design. We focused on observational and experimental MRI studies, and study selection was based on relevance to MRI-derived brain age, age-relevant MRI markers, and dementia-related outcomes. We aim to summarize current evidence linking poor sleep to MRI-derived brain age acceleration and dementia risk, critically appraise proposed mechanistic pathways, and highlight translational gaps that must be addressed before brain age metrics can be implemented in clinical practice.

## 2. Associations and Evidence

### 2.1. Large-Scale Population Studies

Miao et al. analyzed 27,500 UK Biobank participants (mean age, 54.7 years) and constructed composite sleep health scores from five self-reported factors: chronotype, sleep duration (optimal range, 7–8 h), insomnia symptoms, snoring, and daytime sleepiness [5]. Using machine learning applied to 1079 MRI phenotypes, investigators found that participants with poor sleep (≤1 point) exhibited brains 0.99 years older than their chronological age, whereas those with intermediate sleep (2–3 points) exhibited brains 0.62 years older than their chronological age. Each one-point decrease in sleep score is associated with a 0.48-year acceleration in brain age (Table 1). Mediation analysis revealed that systemic inflammation (as measured by C-reactive protein, white blood cell count, platelet count, and granulocyte-to-lymphocyte ratio) explained only 7–10% of the sleep–brain age association, leaving the majority of the association mechanistically unexplained, a critical gap that requires acknowledgment.

**Figure 1:**
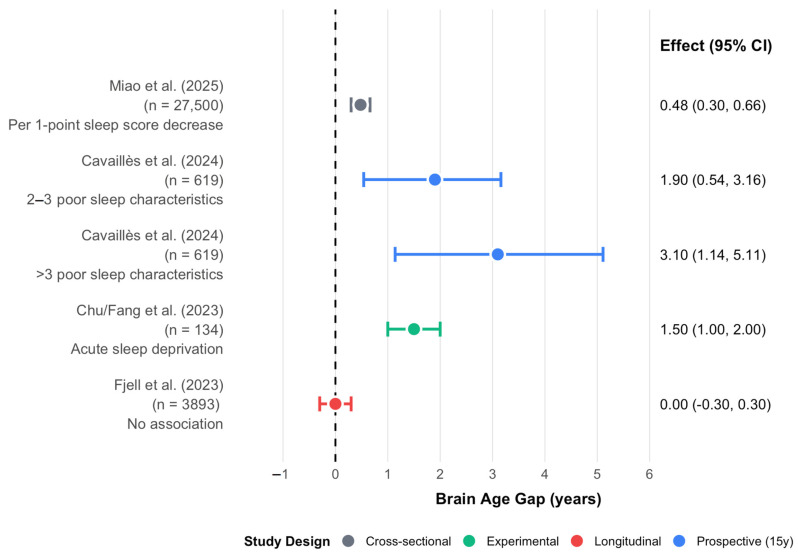
Brain Age acceleration is associated with poor sleep. Forest plot showing effect sizes (years of brain age acceleration) with 95% confidence intervals. Color indicates study design; marker size reflects sample size. The reference line at 0 indicates no effect [5,6,7,8].

The CARDIA prospective cohort enrolled 619 participants, assessed sleep characteristics at a mean age of 40, and conducted brain MRI 15 years later [6]. Those reporting 2–3 poor sleep characteristics showed a 1.9-year (95% CI: 0.54–3.16) greater brain age; those with more than three characteristics demonstrated a 3.1-year (95% CI: 1.14–5.11) acceleration. The 15-year temporal separation strengthens causal inference, though residual confounding from unmeasured lifestyle factors cannot be excluded (Figure 1).

### 2.2. Critical Evaluation of Contradictory Evidence

Not all large-scale investigations confirm these associations. Fjell et al. found no phenotypic or genotypic associations between sleep duration and brain atrophy in 8153 MRI scans from 3893 healthy adults, raising essential questions about publication bias, methodological heterogeneity in brain age algorithms, and potential nonlinear relationships [7]. The discordance between studies employing different brain age estimation approaches (single-modality versus multimodal, and various machine learning architectures) suggests that reported associations may be algorithm-dependent, a fundamental limitation that requires standardization, as shown in Table 1.

**Figure 2:**
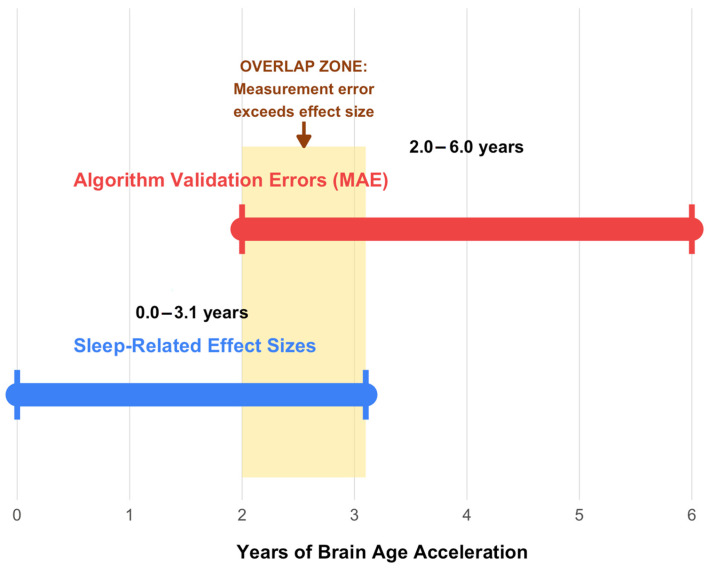
Algorithm validation errors overlap with sleep-related effect sizes. Comparison of brain age algorithm validation errors (mean absolute error (MAE): 2.0–6.0 years, red bar) versus reported sleep effect sizes (0–3.1 years, blue bar). The shaded yellow region indicates the overlap zone where measurement uncertainty equals or exceeds biological effects.

Algorithm heterogeneity substantially complicates interpretation (Table 2). Validation mean absolute errors (2–6 years) are comparable to or exceed the sleep-related effect sizes themselves (1–3 years), raising fundamental questions about whether associations reflect actual biological phenomena or algorithm-specific artifacts (Figure 2).

### 2.3. Experimental Evidence and Reversibility

Fang et al. demonstrated that total sleep deprivation (more than 24 h of wakefulness) increased brain age by 1–2 years in 134 healthy adults across five datasets [8]. Critically, this effect reversed with recovery sleep, suggesting that acute functional rather than permanent structural alterations occurred. This reversibility, while mechanistically informative, cannot be extrapolated to chronic sleep restriction without longitudinal intervention data. The relationship between the effects of acute and chronic sleep loss on brain structure remains poorly characterized (Table 3).

### 2.4. Effect Size Contextualization

While statistically significant in large samples, reported effect sizes merit careful interpretation. A brain age acceleration of 1–3 years represents a relatively modest deviation compared to the ~50-year span of adult brain aging, as shown in Table 1. The clinical significance threshold for the predictive value of BAG magnitude for cognitive decline remains undefined. Furthermore, lifestyle factors, including sleep, account for ≤21% of brain age variance [9], indicating that the majority of brain aging variation arises from factors beyond currently measured sleep parameters.

## 3. Sleep Parameter-Specific Associations with Brain Structure

### 3.1. Distinguishing Sleep-Specific from Shared Mechanisms

Sleep disturbances affect brain structure through mechanisms that range from uniquely sleep-dependent processes to pathways shared with other age-related conditions. Understanding this distinction is critical for identifying the most promising therapeutic targets. Current evidence indicates that sleep fragmentation, sleep stage architecture, and specific aspects of obstructive sleep apnea exhibit sleep-specific neuroanatomical signatures that operate through mechanisms independent of classic neurodegenerative or vascular pathways. In contrast, sleep duration and subjective sleep quality primarily amplify shared vascular, inflammatory, and metabolic pathways common to aging and chronic disease, making it challenging to isolate sleep-specific contributions.

### 3.2. Sleep Fragmentation and Efficiency: The Sleep-Specific Vascular Pathway

**Figure 3:**
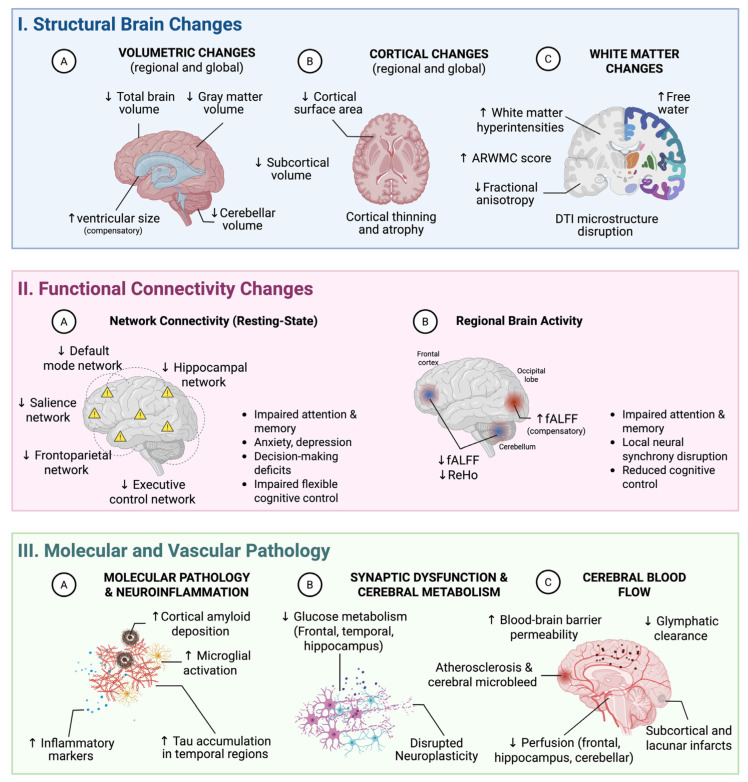
Brain structural, functional, and molecular changes associated with poor sleep. (**I**). Structural brain changes: (**IA**) volumetric changes include decreased total brain, gray matter, and cerebellar volumes, accompanied by compensatory ventricular enlargement; (**IB**) cortical changes consist of regional thinning and reduced surface area, affecting frontal, temporal, and parietal regions, with pronounced atrophy in the hippocampus and precuneus (Alzheimer’s disease-signature regions); (**IC**) white matter changes encompass increased white matter hyperintensities, elevated age-related white matter change (ARWMC) scores, reduced fractional anisotropy, and disrupted diffusion tensor imaging (DTI) microstructure, with increased free water and mean diffusivity throughout major tracts. (**II**) Functional connectivity changes: (**IIA**) resting-state connectivity is reduced within the default mode, hippocampal, salience, frontoparietal, and executive control networks (yellow warning triangles indicate disconnection sites), contributing to impaired attention, memory consolidation, mood regulation, decision-making, and cognitive flexibility; (**IIB**) regional brain activity shows decreased fractional amplitude of low-frequency fluctuation (fALFF) and regional homogeneity (ReHo) in the frontal cortex and cerebellum, with compensatory increases in the occipital lobe (fALFF ↑), indicating disrupted local synchrony and reduced cognitive control. (**III**) Molecular and Vascular Pathology: (**IIIA**) neuroinflammatory changes include increased cortical amyloid deposition, microglial activation, elevated inflammatory markers, and tau accumulation in temporal regions; (**IIIB**) synaptic and metabolic alterations include reduced glucose metabolism in frontal, temporal, and hippocampal areas with impaired neuroplasticity; and (**IIIC**) cerebrovascular changes involve increased blood–brain barrier permeability, microbleeds, reduced perfusion in frontal, hippocampal, and cerebellar regions, impaired glymphatic clearance via perivascular spaces, and subcortical and lacunar infarcts related to small vessel disease ↑: increase; ↓: decrease.

Objective actigraphy consistently demonstrates that lower sleep efficiency and increased wake after sleep onset (WASO) are associated with reduced cortical thickness and hippocampal volume, particularly within medial temporal and parietal regions (Figure 3). These relationships remain independent of amyloid and tau biomarker levels, indicating that sleep fragmentation contributes to neurodegenerative changes through vascular and inflammatory mechanisms rather than direct Alzheimer’s disease (AD) pathology. Autopsy-confirmed neuropathological data of 315 community-dwelling older adults further link higher sleep fragmentation indices to cerebral arteriolosclerosis and subcortical infarcts, establishing a direct mechanistic connection between fragmentation and small-vessel cerebrovascular pathology [4,13,14,15]. Functional consequences appear to precede structural damage: in cognitively normal individuals, higher fragmentation intensity is associated with frontohippocampal hypometabolism and executive dysfunction [16], suggesting that metabolic disruption occurs before visible atrophy and may define a window of opportunity for intervention.

Taken together, this cascade, from sleep disruption to metabolic dysfunction, vascular injury, and structural atrophy, supports sleep fragmentation as a uniquely sleep-dependent vascular pathway rather than merely a correlate of systemic vascular disease. Although only a few studies have directly quantified the MRI-derived brain age gap in relation to fragmentation, the preferential involvement of hippocampal and parietal regions, which heavily weight brain age models, implies that chronic fragmentation is likely to manifest as an older-appearing brain and increased BAG. These fragmentation-related vascular and metabolic changes also overlap with dementia-vulnerable networks, supporting the hypothesis that sleep efficiency and WASO may influence both brain age acceleration and long-term dementia risk, even though causal pathways remain incompletely defined.

### 3.3. Sleep Duration

Large-scale neuroimaging cohorts suggest a U-shaped relationship between sleep duration and brain structure [17,18]. In the UK Biobank (N = 479,420), both short (≤5 h) and long (≥9 h) sleep durations are associated with reduced cortical/subcortical volumes, increased white matter hyperintensities, and decreased fractional anisotropy, with approximately seven hours emerging as the optimal duration for cognitive performance [19]. Quantitative effects are notable: in a cohort of 2334 Hispanic/Latino adults, each additional hour of sleep beyond 9 h was associated with lower total brain and gray matter volumes [20]. These associations persisted for up to 15 years of follow-up and were stronger in older adults with hypertension or low social engagement, underscoring the interactions between sleep duration, vascular health, and cognition [19,21].

White matter appears particularly vulnerable, as duration-related differences in white matter hyperintensities and microstructural injury have been observed in both cross-sectional and longitudinal designs encompassing more than 26,000 participants [22]. These findings, together with large cohorts showing widespread cortical and subcortical atrophy at extreme durations and at least one major longitudinal null study, are summarized in Table 4. However, the U-shaped pattern is not entirely consistent; optimal duration ranges from 6 to 8 h across studies, and at least one investigation involving 8153 MRI scans reported no phenotypic or genotypic association between sleep duration and brain atrophy [7]. Collectively, these data support the view that very short and very long sleep may act as markers of broader vascular or metabolic dysfunction rather than reflecting purely sleep-specific neurotoxicity.

Direct analyses of MRI-derived brain age gap as a function of sleep duration remain limited. Nevertheless, the widespread cortical and white matter abnormalities associated with extreme durations occur in regions that strongly influence brain age estimates, suggesting that persistently short or long sleep is likely to be reflected in higher BAG in at least some individuals. These duration-related structural changes also overlap with networks vulnerable to dementia, implying that sleep duration may contribute to both brain age acceleration and subsequent dementia risk, even though causality and clinically meaningful thresholds for BAG remain to be established.

### 3.4. Insomnia and Subjective Sleep Quality

Self-reported poor sleep quality, insomnia symptoms, and sleep dissatisfaction are consistently associated with accelerated brain aging and reduced cortical volume across multiple cohorts. These relationships appear to be mediated by several biological pathways, including systemic inflammation (e.g., C-reactive protein), hypothalamic–pituitary–adrenal axis dysregulation, and comorbid anxiety and depression, each of which independently influences brain structure. Mediation analyses indicate that inflammatory markers account for only a modest fraction (approximately 7–10%) of the association between sleep disturbance and MRI-derived brain age, leaving most of the variance mechanistically unexplained [5].

In clinical populations, such as individuals with late-onset depression, insomnia symptoms, and altered sleep quality, sleep alterations confer an additive risk; polysomnography shows that reductions in slow-wave and REM sleep, and in K-complex density, are associated with impaired executive function and memory, whereas higher proportions of light N2 sleep are associated with cortical thinning. Conversely, greater amounts of deep sleep appear neuroprotective, highlighting the importance of sleep stage quality beyond total sleep duration [27,28]. At the same time, methodological limitations warrant caution: the majority of brain age studies rely on self-reported sleep metrics, which are often discordant with objective measures such as polysomnography and actigraphy, potentially inflating or distorting effect size estimates and making it difficult to disentangle true physiological sleep effects from reporting biases related to mood, personality, and recall (Supplementary Table S1).

Direct evidence linking insomnia or subjective sleep quality to MRI-derived brain age differences remains limited, with most studies focusing on regional atrophy or cortical thickness rather than formal brain age models [23]. Nevertheless, the preferential involvement of frontal and temporal cortices, together with stress- and inflammation-related mechanisms, suggests that chronic insomnia and poor perceived sleep quality are likely to contribute to an older-appearing brain in at least some individuals [5]. Because these same regions and networks are central to dementia pathophysiology, insomnia-related changes may influence both BAG and long-term dementia risk, although stronger longitudinal and intervention data are needed to establish causal pathways and clinically relevant thresholds.

### 3.5. Sleep Architecture: Stage-Specific Vulnerability

Polysomnography-based assessments reveal distinct regional vulnerability patterns across specific sleep stages, providing some of the strongest evidence that sleep microarchitecture exerts effects on the brain beyond total duration or global sleep quality. These stage-dependent associations show regional specificity that is not readily explained by other vascular or metabolic risk factors, supporting the view that sleep architecture is a unique determinant of brain structure. In the Framingham Heart Study Offspring cohort (N = 492, 17-year follow-up), even small reductions in slow-wave sleep (SWS) were associated with substantial decreases in total brain volume and pronounced vulnerability of the frontal cortex, underscoring the importance of deep sleep for maintaining cortical integrity [29].

REM-related metrics also show anatomically targeted effects. In the ARIC cohort (N = 271), a lower percentage of REM sleep was linked to smaller volumes in AD-signature regions such as the inferior parietal lobule and precuneus [29], while increased REM latency independently predicted AD-pattern cortical thinning and higher white matter hyperintensity burden in a larger, diverse sample (N = 842) [28]. Sleep spindle markers, including K-complex density, correlate positively with cingulate cortex thickness in patients with Alzheimer’s disease, suggesting that preserved spindle activity may protect cortical structure through thalamocortical synchronization and memory consolidation processes [30].

The stage-specific nature of these associations is further highlighted by opposing effects of light versus deep sleep: higher proportions of N2 (light) sleep are associated with temporal and parietal cortical thinning, whereas higher proportions of deep sleep show protective effects in the same regions [24]. This pattern supports a model in which altered sleep microarchitecture, rather than simply short or long sleep, contributes to regionally specific neurodegeneration. Although formal analyses of MRI-derived brain age gap for SWS, REM, and spindle metrics are still scarce, the preferential involvement of frontal and parietal association cortices strongly suggests that adverse changes in sleep architecture would be captured as higher BAG in brain age models. Because these same regions are central to dementia-related atrophy patterns, stage-specific disturbances in SWS and REM are likely to influence both brain age acceleration and dementia risk, making sleep-stage–targeted interventions a particularly promising neuroprotective strategy.

### 3.6. Obstructive Sleep Apnea (OSA)

OSA severity, typically quantified by apnea–hypopnea index (AHI), is associated with reduced gray matter concentration in frontal, cingulate, thalamic, and hippocampal regions, together with widespread white matter injury in patients with moderate–severe disease [24,31]. REM-predominant OSA shows additional, stage-specific vulnerability of subcortical white matter, where REM-related respiratory events predict greater white matter hyperintensity burden, highlighting the interaction between sleep stage, intermittent hypoxia, and vascular injury [24]. Importantly, structural abnormalities often show only partial reversibility despite adequate treatment. Even among patients adherent to continuous positive airway pressure (CPAP) for at least 30 days with ≥6 h per night, a substantial subset (approximately 30%) demonstrates persistent elevations in mean diffusivity across nearly one-fifth of assessed white matter tracts, indicating incomplete recovery of microstructural integrity. These findings suggest that chronic OSA may leave a residual “vascular–hypoxic scar” in both gray and white matter despite symptomatic improvement [24,31,32].

Direct application of MRI-derived brain age models to OSA cohorts remains limited, but the preferential involvement of frontal, limbic, and hippocampal circuitry, regions that heavily influence brain age estimates, strongly implies that moderate–severe OSA is likely to present as an older-appearing brain with an increased BAG. Given that these same regions and tracts are central to dementia-related atrophy and disconnection, OSA-related structural injury may contribute to both brain age acceleration and elevated dementia risk, reinforcing the importance of early detection and sustained treatment to mitigate long-term neurobiological consequences.

### 3.7. Sex-Specific Associations

Emerging evidence demonstrates that the relationship between sleep parameters and brain structure exhibits significant sex-specific patterns that vary across the lifespan. In young and middle-aged adults, poor sleep quality is associated with reduced gray matter volume in females but not males, with particular vulnerability observed in the hippocampus, parahippocampal gyrus, inferior parietal lobule, and inferior temporal gyrus [28,33]. Conversely, among older adults, males demonstrate greater susceptibility to white matter microstructural deterioration associated with poor sleep, including reduced neurite density, decreased restricted isotropic diffusion, and increased amygdala free water fraction [34]. In obstructive sleep apnea, females exhibit more severe white matter injury and greater cognitive impairment compared to males with equivalent disease severity, suggesting sex-specific pathophysiological mechanisms related to hormonal influences on breathing control, upper airway anatomy, and inflammatory responses [35]. These findings underscore the importance of considering sex as a biological moderator when evaluating sleep-related brain changes and developing targeted interventions for brain health across the lifespan.

## 4. Functional Connectivity and Regional Activity Disruption

### 4.1. Network-Level Alterations

Resting-state functional MRI (fMRI) meta-analyses demonstrate widespread network disruption following sleep deprivation and chronic sleep disorders [36,37]. The default mode network (DMN), comprising posterior cingulate cortex, precuneus, medial prefrontal cortex, and angular gyrus, shows reduced within-network connectivity and altered anti-correlations with task-positive networks in both acute sleep deprivation and OSA, and these changes are linked to impaired memory consolidation and attention (Figure 3) [38,39,40]. In adolescents, more regular sleep patterns correlate with stronger DMN connectivity, supporting a dose–response relationship between sleep regularity and network integrity [41].

Alterations are not restricted to the DMN. Salience network connectivity between the anterior insular and dorsal anterior cingulate cortex decreases in insomnia and poor subjective sleep quality, impairing stimulus filtering and network switching between the DMN and executive systems and thereby linking sleep disruption to anxiety, depression, and emotional dysregulation [42]. Executive control network connectivity between the dorsolateral prefrontal and posterior parietal cortices is reduced in both resting-state and task-based paradigms, with the severity of disconnection predicting working memory and decision-making deficits. The overlapping frontoparietal network shows similar vulnerability, with a functional disruption scaling with the duration of sleep deprivation [43,44].

Hippocampal-centered networks represent a critical pathway to cognitive decline [45]. Sleep disruption reduces hippocampal–prefrontal and hippocampal–parietal connectivity, undermining memory encoding and retrieval [46,47]. In older adults with chronic sleep disorders, progressive hippocampal–prefrontal disconnection tracks with accelerated cognitive aging and higher dementia vulnerability, suggesting that network-level changes may facilitate neurodegenerative cascades [48,49,50]. Although most of these studies do not directly apply MRI-derived brain age models, the affected networks substantially overlap with those that drive BAG estimates, implying that sleep-related network disconnection is likely to manifest as functional correlates of brain age acceleration and contribute to dementia risk.

### 4.2. Regional Activity Alterations

OSA and sleep deprivation also alter spontaneous neural activity, as measured by multiple fMRI-derived metrics [25,51,52]. Fractional amplitude of low-frequency fluctuation analyses shows decreased activity in the frontal cortex and cerebellum, together with paradoxical increases in the occipital regions in severe OSA, potentially reflecting compensatory shifts in sensory processing [1]. Regional homogeneity (ReHo), indexing local neural synchrony, is reduced in cerebellar and frontal regions, indicating impaired coordination within localized circuits [53]. The ALFF reductions across cognitive control and memory-related areas parallel fALFF and ReHo findings, supporting the concept that healthy sleep is required to maintain both large-scale connectivity and local circuit synchronization [54].

These regional activity changes map onto cortical and subcortical hubs that substantially contribute to brain age predictions in structural and multimodal MRI models [55]. While most existing studies report fMRI outcomes rather than explicit BAG metrics, convergence of reduced activity in frontal, cerebellar, and hippocampal networks with structural alterations in the same regions suggests that sleep-related disruptions in regional activity are likely to co-occur with higher MRI-derived brain age [56]. Given that these regions are central to cognitive control and memory, such functional alterations may represent early, potentially reversible correlates of brain age acceleration and increased dementia risk before the emergence of overt atrophy [57,58,59,60].

### 4.3. Clinical Implications and Limitations

Functional network and regional activity disruption creates system-wide dysregulation that extends beyond isolated structural lesions. Alterations in the DMN are linked to impaired autobiographical memory; salience network dysfunction undermines emotional regulation and adaptive switching between internal and external focus; and fragmentation of executive and frontoparietal networks degrades cognitive control, decision-making, and working memory [61]. Hippocampal disconnection further threatens the integrity of episodic memory systems and appears to track with accelerated cognitive aging in individuals with chronic sleep disturbance [62].

Critically, functional connectivity and activity changes may precede overt structural atrophy, making them attractive candidates as early markers and intervention targets [8]. Experimental data showing that brain age acceleration induced by acute sleep deprivation can reverse with recovery sleep support the notion that at least part of the functional and structural “brain age cost” of poor sleep is modifiable, particularly at earlier stages [8,63]. However, most evidence is cross-sectional, limiting causal inference, and neurodegenerative processes may themselves disrupt sleep-regulatory nuclei, creating bidirectional feedback between sleep and brain changes. Heterogeneity in fMRI acquisition, preprocessing, and analytic pipelines further complicates synthesis and may partly explain variability in reported effect sizes across cohorts [64].

For clinical translation, it will be essential to standardize functional connectivity methods, integrate fMRI measures with MRI-derived brain age models, and validate combined markers against longitudinal cognitive outcomes and incident dementia. Such work will clarify whether sleep-related functional network disruptions and elevated BAG provide additive or synergistic information for risk stratification and whether modifying sleep can meaningfully alter brain age trajectories and dementia risk in real-world populations.

### 4.4. Key Points Related to Functional Connectivity and Regional Activity

- Sleep deprivation and chronic sleep disorders disrupt large-scale networks (DMN, salience, executive, frontoparietal, hippocampal), in patterns that align with cognitive decline and dementia vulnerability.
- These network and regional activity changes affect frontal, parietal, hippocampal, and cerebellar hubs that substantially contribute to MRI-derived brain age estimates, implying that functional disconnection is likely to accompany or amplify brain age acceleration.
- Functional connectivity and activity alterations often emerge before overt structural atrophy and may be at least partially reversible with sleep restoration, suggesting a window in which interventions could modify both BAG and future dementia risk.
- Cross-sectional designs and heterogeneous fMRI methodologies currently limit causal inference and comparability; standardized functional protocols integrated with brain age models and longitudinal cognitive outcomes are needed to define the clinical utility of these markers.

## 5. Mechanisms

Despite the identification of several biological pathways, most of the association between poor sleep and MRI-derived brain age remains mechanistically unexplained, limiting the precision of therapeutic targeting (Table 5). Current data point to converging roles for glymphatic impairment, neuroinflammation, vascular injury, and synaptic–metabolic dysfunction, but their relative contributions to brain age acceleration and dementia risk are still not well quantified.

### 5.1. Neuroinflammation

Sleep restriction experimentally increases inflammatory markers such as interleukin-6 (IL-6), tumor necrosis factor-α (TNF-α), and C-reactive protein (CRP) and activates microglia in animal and human studies. In a large cohort, mediation analysis showed that systemic inflammatory markers explained only modest portion (7–10%) of the association between composite sleep scores and MRI-derived brain age, indicating that inflammation is an important but partial contributor to sleep-related brain age acceleration [5]. The precise inflammatory pathways, whether peripheral cytokines cross the blood–brain barrier, trigger central neuroinflammation, or act indirectly via vascular or metabolic routes, remain incompletely characterized [5].

From a brain age perspective, these findings suggest that targeting systemic inflammation may attenuate only a small fraction of sleep-related BAG, and that additional mechanisms must be addressed to meaningfully modify brain age trajectories. Given that chronic low-grade inflammation is also implicated in dementia pathogenesis, sleep-related inflammatory changes may contribute jointly to higher BAG and elevated dementia risk, but their independent and combined effects require further longitudinal study.

### 5.2. Glymphatic System Dysfunction

Converging evidence supports a role for sleep in regulating brain waste clearance via the glymphatic system, a perivascular network that facilitates cerebrospinal fluid-interstitial fluid exchange and removal of metabolites such as amyloid-β and tau [4,70]. Experimental work has shown that neuromodulatory changes during non-REM sleep drive coordinated fluctuations in cerebral blood volume and CSF flow [71], and that sleep, compared with wakefulness, reduces CSF concentrations of amyloid-β and tau in randomized crossover designs [13].

In humans, direct imaging of glymphatic function remains technically challenging. The DTI-ALPS index provides an indirect MRI-based proxy of glymphatic activity by quantifying water diffusivity along perivascular spaces, with lower values interpreted as impaired clearance; however, validation against gold-standard clearance measures is still limited [72]. Despite this, DTI-ALPS studies consistently show reduced glymphatic indices in insomnia, OSA, and poor sleep quality, with dose–response relationships (worse sleep → lower ALPS) and partial reversibility as sleep improves, and exacerbated glymphatic impairment when sleep disorders coexist with Alzheimer’s disease (Table 5).

These findings are highly relevant to brain age because impaired clearance of amyloid-β, tau, and other metabolites may accelerate structural and microstructural changes that are captured by BAG models. Nonetheless, it remains unclear whether glymphatic dysfunction is a primary driver of brain age acceleration or an epiphenomenon of broader vascular and neurodegenerative processes. Future mechanistic studies using advanced imaging (e.g., contrast-enhanced MRI or PET clearance tracers) and longitudinal brain age modeling will be essential to define the causal contribution of glymphatic failure to BAG and dementia risk.

### 5.3. Vascular Mechanisms

Sleep disorders are strongly linked to hypertension, endothelial dysfunction, and small vessel cerebrovascular disease. White matter hyperintensities, a radiological marker of small-vessel injury, are more prevalent with REM-predominant OSA and with adverse sleep behaviors, and are themselves associated with accelerated brain aging and cognitive decline [69]. Autopsy studies further show that higher sleep fragmentation is associated with greater arteriolosclerosis and subcortical infarcts, providing neuropathological confirmation of a sleep–vascular pathway [22].

Experimental sleep restriction can acutely increase blood pressure and impair endothelial function, but it is not yet clear to what extent these short-term hemodynamic changes translate into chronic structural damage captured by brain age models [73,74]. Existing longitudinal data suggest that multiple sleep parameters contribute to WMH burden and white matter microstructural injury, but the proportion of sleep-related BAG that is mediated specifically through vascular mechanisms remains unquantified [22,75]. Clarifying this will require prospective studies that integrate sleep phenotyping, vascular biomarkers, detailed WMH and DTI measures, and serial brain age estimates.

### 5.4. Synaptic and Metabolic Pathways

Sleep supports synaptic homeostasis, including downscaling and pruning, which are essential for maintaining neural efficiency and preventing synaptic overload [76]. Chronic sleep restriction is also associated with insulin resistance, altered glucose metabolism, and other metabolic disturbances that are established risk factors for cognitive decline and structural brain changes. However, direct human evidence linking these synaptic and metabolic processes to MRI-detectable brain age acceleration is still lacking.

The hypothesized mechanistic chain, from sleep loss to synaptic dysfunction, metabolic dysregulation, network inefficiency, and finally to macrostructural changes detectable on conventional MRI, has not yet been fully delineated. Multimodal studies integrating functional imaging, metabolic and neurodegenerative biomarkers, and post-mortem analyses with longitudinal BAG modeling will be required to determine how much of sleep-related brain age acceleration is driven by synaptic and metabolic pathways versus vascular, inflammatory, or glymphatic processes.

### 5.5. Key Points (Mechanisms)

- Glymphatic, inflammatory, vascular, and synaptic–metabolic pathways each contribute to sleep-related brain changes, but together explain only a minority of the observed association between poor sleep and MRI-derived brain age.
- DTI-ALPS and CSF biomarker studies provide converging evidence that sleep enhances glymphatic clearance of amyloid-β and tau, suggesting a plausible route by which inadequate sleep could accelerate brain aging and dementia pathology.
- Vascular injury, reflected in WMH burden, arteriolosclerosis, and microstructural white matter damage, is tightly linked to adverse sleep patterns and likely mediates part of the sleep–BAG association, particularly for fragmentation and OSA.
- Synaptic and metabolic mechanisms are strongly implicated by experimental and epidemiological data, but require integrated multimodal and longitudinal brain age studies to quantify their specific contributions to brain age acceleration and dementia risk.

## 6. Clinical Implications

### 6.1. Risk Stratification

Brain age has the potential to enhance dementia risk prediction when combined with genetic factors (e.g., APOE ε4) and fluid biomarkers [77]. However, lifestyle and sleep factors together explain only a modest proportion of brain age variance (≤21%), and the BAG threshold that constitutes clinically meaningful risk remains uncertain [78]. A 1–3-year increase in BAG is statistically associated with adverse outcomes, but its predictive value for incident dementia, including sensitivity, specificity, and clinically useful cut-points, requires validation in prospective cohorts with adjudicated diagnoses.

### 6.2. Intervention Trials

To date, no randomized controlled trials have directly tested whether cognitive behavioral therapy for insomnia (CBT-I) or sleep extension protocols modify MRI-derived brain age trajectories (Table 3). CPAP studies in OSA suggest that structural reversibility is possible but slow: near-complete white matter recovery may require up to 12 months of sustained treatment, and approximately 30% of adherent patients exhibit incomplete structural response. Experimental data showing that acute sleep deprivation-related brain age acceleration reverses with recovery sleep support a degree of plasticity, but the long-term effects of chronic sleep loss may differ fundamentally and remain poorly characterized. Without interventional trials that include serial MRI and brain age measures as key outcomes, recommendations to use sleep optimization specifically as a brain age-targeted neuroprotective strategy remain preliminary.

### 6.3. Public Health Implications

Given the 30–60% of middle-aged adults report inadequate sleep patterns, population-level sleep interventions could, in principle, have substantial impact on brain health. However, translating observational associations between sleep, BAG, and dementia into public health policy requires demonstration that improving sleep causally modifies brain age trajectories, reduces dementia incidence, and does so in a cost-effective manner. Such causal validation, intervention efficacy data, and formal health economic analyses are currently lacking.

## 7. Critical Gaps and Limitations

### 7.1. Methodological Limitations Requiring Resolution

Most available evidence is derived from cross-sectional or retrospective analyses, which fundamentally limit causal inference and cannot exclude bidirectional relationships in which neurodegenerative processes themselves disrupt sleep-regulatory nuclei (Supplementary Table S1). Most studies rely on self-reported sleep measures that are vulnerable to measurement error and recall bias, and well-documented discordance between subjective and objective sleep (polysomnography, actigraphy) is rarely addressed at scale.

Heterogeneity in brain age estimation methods further constrains reproducibility and clinical translation. Algorithms differ widely in architecture, training datasets, MRI modalities, and preprocessing pipelines, and different models applied to the same cohort can yield divergent conclusions, as illustrated by null atrophy-based findings in some studies versus positive BAG associations in others. Fewer than 1 in 5 brain age algorithms have been validated against clinical endpoints, such as dementia incidence or cognitive decline, limiting confidence in their utility as prognostic tools [7].

Generalizability is also restricted. Much of the literature depends on the UK Biobank, which predominantly includes White European, relatively healthy, and highly educated participants, introducing selection and healthy-volunteer biases (Table 6) [79]. Only a small number of cohorts (e.g., HABS-HD, SOL-INCA, CARDIA) provide meaningful ethnic and socioeconomic diversity, and virtually no large-scale studies from Asian, African, Middle Eastern, or South American populations are available. Because sleep patterns, sleep-disorder prevalence, and brain-aging trajectories vary across populations, current evidence cannot determine whether sleep–BAG associations generalize beyond European-ancestry cohorts. This lack of diversity substantially limits the causal generalizability of current sleep–BAG findings and represents a major barrier to clinical translation.

### 7.2. Mechanistic Gaps Limiting Translational Potential

Even in studies that include inflammatory markers, most of the association between sleep disturbance and brain age remains mechanistically unexplained, indicating that currently measured pathways account for only a fraction of the observed effects [80]. The relative contributions of glymphatic dysfunction, small-vessel disease, metabolic disruption, and synaptic alterations need to be delineated through multimodal designs that combine MRI-derived brain age with PET imaging, CSF and plasma biomarkers, and high-resolution sleep phenotyping [81]. Critical temporal windows during which sleep exerts maximal neuroprotective effects are also undefined; it is unknown whether risk accumulates linearly, shows thresholds, or follows nonlinear dose–response patterns, information that would directly inform when and how aggressively to intervene [82,83].

Acute sleep deprivation studies show that short-term MRI-predicted brain age changes can reverse with recovery sleep. Still, it is unclear whether chronic sleep loss leads to partially irreversible structural alterations or remains amenable to correction [8]. This distinction has immediate therapeutic implications for determining whether interventions can normalize BAG in individuals with long-standing sleep disorders.

### 7.3. Clinical Translation Barriers

At present, no randomized controlled trials have demonstrated that improving sleep consistently alters brain age trajectories, representing a primary barrier to clinical adoption (Table 3). The BAG magnitude that should trigger clinical concern, the degree of BAG reduction required for meaningful benefit, and the practical relevance of observed 1–3-year effect sizes all remain undefined. Moreover, economic evaluations of MRI-based brain age screening integrated with sleep assessment or dementia risk workflows are lacking, making it difficult to justify implementation in routine care.

### 7.4. Future Research Priorities and Roadmap

**Figure 4:**
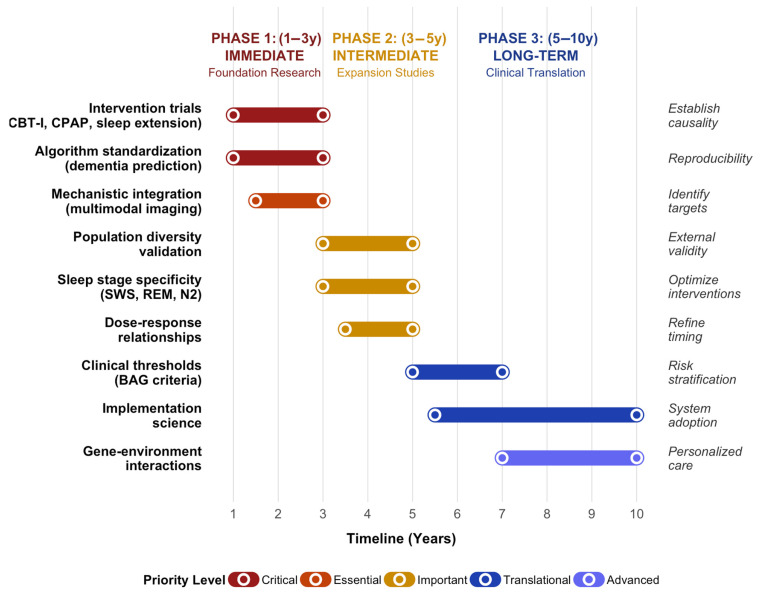
Research priorities roadmap for establishing sleep as an evidence-based neuroprotective intervention. Gantt-style timeline illustrating sequential and parallel research priorities across three phases. Horizontal bars represent the duration of each priority and are color-coded by urgency (dark red: critical priorities; orange: essential mechanistic work; gold: important expansion studies; dark blue: translational research; light blue: advanced personalized medicine approaches). Phase 1 (years 1–3) focuses on RCTs of CBT-I, CPAP, and sleep extension with serial MRI-based brain age outcomes; development and standardization of BAG algorithms benchmarked against dementia and cognitive decline; and mechanistic multimodal studies incorporating PET, CSF, plasma biomarkers, and emerging glymphatic imaging. Phase 2 (years 3–5) includes validation in non-European and socioeconomically diverse populations, sleep-stage-specific experiments (SWS, REM, N2), and detailed dose–response modeling. Phase 3 (years 5–10) involves defining clinically actionable BAG thresholds, integrating brain age into personalized risk stratification, conducting health economic analyses, and testing workflow integration in neurology and sleep clinics. BAG, brain age gap; CBT-I, cognitive behavioral therapy for insomnia; CPAP, continuous positive airway pressure; N2, stage 2 non-REM sleep; REM, rapid eye movement; SWS, slow-wave sleep.

Establishing sleep optimization as an evidence-based neuroprotection strategy requires a staged research roadmap that spans immediate foundational work through long-term clinical implementation (Figure 4). Phase 1 (years 1–3) emphasizes randomized trials of sleep interventions with serial MRI, algorithm standardization, and validation against dementia outcomes, and mechanistic multimodal studies. Phase 2 (years 3–5) prioritizes expansion into diverse populations, sleep-stage-specific studies, and detailed characterization of dose–response and critical windows. Phase 3 (years 5–10) focuses on defining clinical thresholds for BAG, integrating brain age metrics into risk models, conducting cost-effectiveness analyses, and applying implementation science frameworks for real-world deployment.

### 7.5. Essential Immediate Priorities

The first phase (years 1–3) should prioritize causal evidence and methodological standards. Randomized controlled trials are needed to determine whether CBT-I, CPAP, and structured sleep extension can modify brain age trajectories, using serial MRI-derived BAG as a primary or key secondary outcome to quantify reversibility and biological efficacy. These trials will likely require multisite collaboration and substantial funding, but are indispensable for moving from association to causation.

In parallel, standardized brain age algorithms must be developed and validated across imaging platforms and populations, with performance benchmarked against dementia incidence and cognitive decline. Longitudinal mechanistic studies integrating BAG with PET amyloid, tau, and inflammatory tracers, CSF biomarkers, and glymphatic imaging will be crucial for dissecting biological pathways. Expanding population diversity and emphasizing objective sleep measures (e.g., polysomnography or validated wearables) will address current limitations in self-reported sleep and limited generalizability.

### 7.6. Advanced Mechanistic Questions

The second phase (years 3–5) should deepen mechanistic and population-specific understanding. High-density EEG–MRI studies can help identify which sleep stages (e.g., SWS, REM, N2) are most critical for preserving structural and functional integrity, and clarify how stage-specific disruptions map onto BAG and dementia risk. Refining dose–response and nonlinear models will help define safe and high-risk ranges of sleep duration and quality. Identifying developmental and aging windows of maximal vulnerability, and elucidating gene–environment interactions that modulate susceptibility to sleep-related brain aging will be essential for targeted prevention strategies.

### 7.7. Clinical Implementation Research

The third phase (years 5–10) focuses on clinical integration. Prospective studies should establish clinically meaningful BAG thresholds linked to dementia incidence and cognitive decline, enabling BAG-guided timing of interventions and risk communication. Integrated models combining brain age with genetic, biomarker, and lifestyle data may improve individualized risk stratification and treatment prioritization. Health economic analyses are needed to determine the cost-effectiveness of incorporating MRI-derived brain age into routine care, particularly when paired with sleep assessment and targeted interventions. Finally, implementation-science approaches should be used to pilot and optimize brain age-based workflows in neurology, psychiatry, and sleep medicine, ensuring that advances in imaging biomarkers translate into tangible clinical benefit.

## 8. Conclusions

Converging observational evidence links inadequate sleep to modest MRI-derived brain age acceleration of 1–3 years, with parallel associations with structural and functional brain changes relevant to dementia risk. Mechanistic pathways involving neuroinflammation, glymphatic dysfunction, and small-vessel vascular injury provide biological plausibility. Yet, most of the sleep–brain age relationship remains mechanistically unexplained, and the long-term impact of chronic sleep disturbance on brain age trajectories is still uncertain.

Several critical gaps currently limit clinical translation. These include the absence of randomized intervention trials demonstrating that improving sleep modifies brain age, the predominance of cross-sectional designs that preclude firm causal inference, heavy reliance on self-reported sleep measures, substantial heterogeneity in brain age algorithms, and limited population diversity in existing cohorts. Although reported effect sizes are statistically robust, they are modest, and clinically meaningful thresholds for BAG have not been defined.

Sleep remains a theoretically modifiable target for preserving brain health and potentially reducing dementia risk, but establishing it as an evidence-based neuroprotective strategy will require rigorous causal validation. Priority areas include randomized trials of sleep interventions with serial brain age outcomes, standardized and clinically validated BAG algorithms, deeper mechanistic elucidation using multimodal approaches, and replication in ethnically and socioeconomically diverse populations. Only by addressing these gaps can sleep optimization move from a promising hypothesis to an implemented clinical tool for brain age modification and dementia prevention. Taken together, these limitations indicate that the current translational readiness of MRI-derived brain age metrics for routine clinical use remains low to moderate.

## Figures and Tables

**Table 1:** Large-scale studies of sleep and MRI-derived brain age.

Study	Year	Population (N)	Sleep Assessment	Brain Age Method	Brain Age Gap Finding	Effect Size
Miao et al. [5]	2025	UK Biobank (27,500; age 54.7 ± 8 y)	Composite score: chronotype, duration, insomnia, snoring, sleepiness	ML on 1079 MRI phenotypes	Poor sleep (≤1 pt): +0.99 y; Intermediate (2–3 pts): +0.62 y	0.48 y per point decrease; Inflammation mediates 7–10%
Cavaillès et al. [6]	2024	CARDIA (619; age 40 → 55 y, 15 y follow-up)	Self-report: 6 characteristics	ML-based brain age	2–3 poor sleep characteristics: +1.9 y (0.54–3.16); >3: +3.1 y (1.14–5.11)	Prospective design strengthens causality
Fjell et al. [7]	2023	Lifebrain (8153 MRIs; 3893 adults)	Self-report duration	Longitudinal atrophy trajectories	No association (phenotypic or genetic)	Cross-sectional U-shape: 6.5 h optimal
Chu et al. [8]	2023	Multi-site (134; age 19–39 y)	Total sleep deprivation (>24 h)	T1-weighted prediction model	Acute TSD: +1–2 y BAG	Reversible with recovery sleep

BAG, brain age gap; ML, machine learning; TSD, total sleep deprivation. Effect sizes represent the number of years of apparent brain aging associated with poor sleep; y, years.

**Table 2:** Brain age algorithm heterogeneity across studies.

Study	Algorithm Architecture	Training Dataset	N (Training)	Age Range (Training)	MRI Modalities	Preprocessing Pipeline	Validation MAE	Clinical Outcome Validation
Li et al. (2025) [1]	Deep learning approach	UK Biobank + validation cohorts	Large sample	Middle-aged to older adults	T1-weighted, DTI, other	Deep neural network	2.4–2.5 y	Validated: predicts mortality
Cole et al. (2018) [2]	Gaussian Processes Regression	Multiple cohorts	>2000	18–90 y	T1-weighted	SPM-based features	~5 y	Validated: BAG predicts mortality
Miao et al. (2025) [5]	Machine learning (not specified)	1079 MRI phenotypes	UK Biobank subset	40–69 y	Multimodal features	UK Biobank pipeline	Not reported	Not validated against dementia incidence
Cavaillès et al. (2024) [6]	Machine learning on T1 MRI	CARDIA-specific	619	40–55 y	T1-weighted	FreeSurfer-based	Not reported	Not validated against clinical outcomes
Fjell et al. (2023) [7]	Longitudinal atrophy trajectories	Lifebrain cohort	3893 (8153 scans)	Adult range	T1-weighted	FreeSurfer longitudinal pipeline	Not brain age (atrophy-based)	NULL FINDING for sleep-atrophy
Chu/Fang et al. (2023) [8]	T1-weighted prediction model	Multisite validation	134	19–39 y	T1-weighted	Standardized across sites	~2.4–2.5 y	Not applicable (acute study)
General deep learning approaches	CNN, ResNet, 3D-CNN architectures	Variable (IXI, UK Biobank, ADNI, others)	1000–50,000+	Variable	T1-weighted primarily, some multimodal	Highly variable (FSL, FreeSurfer, SPM, custom)	2–6 y	Rarely validated against dementia incidence

MAE, mean absolute error (lower is better accuracy); CNN, convolutional neural network; DTI, diffusion tensor imaging; SPM, Statistical Parametric Mapping; ADNI, Alzheimer’s Disease Neuroimaging Initiative; y, years.

**Table 3:** Sleep intervention studies: effects on brain structure.

Intervention	Study (Year)	Design	N	Duration	MRI Outcome	Main Finding	Reversibility
CPAP for OSA							
Sleep recovery	Chu et al. (2023) [8]	Experimental	134 young adults	Post-TSD	Brain age model	TSD + 1–2 y BAG; reversed with recovery	Yes (acute)
CPAP	Castronovo et al. (2014) [9]	Prospective	17 severe OSA vs. 15 controls	3 and 12 months	DTI white matter	3 mo: limited WM recovery; 12 mo: near-complete reversal + cognitive gains	Yes (requires 12 mo)
CPAP	Maresky et al. (2019) [10]	Prospective	7 OSA	6 weeks	DTI + perfusion	↑ FA, ↑ CBF (hippocampus, temporal); ↓ MD	Partial (short-term)
CPAP	Liu et al. (2022) [11]	Prospective	20 severe OSA	3 months	DTI-TBSS	No significant WM changes despite clinical improvement	No (at 3 mo)
CPAP	Xiong et al. (2017) [12]	Prospective	29 OSA (≥6 h/night adherent)	≥30 days	DTI-TBSS	30% residual sleepers: persistent ↑ MD in 17.8% WM tracts	Incomplete in the subset
Sleep Manipulation							
Sleep vs. Deprivation	Lyckenvik et al. (2025) [13]	RCT crossover	Healthy adults	Single night	CSF Aβ and tau	Sleep reduces CSF Aβ and tau	Acute effect
CBT-I	—	—	—	—	—	No published RCTs with MRI outcomes	Unknown

Aβ, amyloid-beta; BAG, brain age gap; CBF, cerebral blood flow; CPAP, continuous positive airway pressure; CSF, cerebrospinal fluid; DTI, diffusion tensor imaging; FA, fractional anisotropy; MD, mean diffusivity; OSA, obstructive sleep apnea; RCT, randomized controlled trial; TBSS, tract-based spatial statistics; TSD, total sleep deprivation; WM, white matter; CBT-I: cognitive behavioral therapy for insomnias; y: years; mo: months; ↑: increase; ↓: decrease.

**Table 4:** Sleep parameter-specific neurostructural signatures.

Study (Year)	Sleep Parameter	N	Assessment Method	MRI Findings	Brain Regions Affected	Clinical Correlation
DURATION						
Tai et al., 2022 [19]	Short (≤5 h) and Long (≥9 h)	479,420	Self-report	↓ Cortical/subcortical volumes; ↑ WMH; ↓ FA	Global, widespread	U-shaped; 7 h optimal for cognition
Gonzalez et al., 2024 [20]	>9 h	2334	Self-report	↓ Total brain (β = −0.05/h); ↓ GM (β = −0.17/h)	Global, gray matter	Hispanic/Latino cohort; dose–response
Wang et al., 2024 [21]	Short/Long	Large UK Biobank	Self-report	↓ Cortical/subcortical volumes	Multiple regions	Cross-sectional and longitudinal
Fjellet al., 2023 [7]	Duration (general)	8153 MRIs	Self-report	No association with atrophy	N/A	NULL FINDING—no phenotypic/genetic link
Ning et al., 2023 [22]	Duration + WM	26,354	Self-report + DTI	↑ WMH; microstructural injury	White matter, global	Cross-sectional and longitudinal validation
FRAGMENTATION						
André et al., 2019 [16]	↑ SF intensity	66	Actigraphy	Mediates hypometabolism → executive dysfunction	Frontohippocampal	Cognitively normal only
Lim et al., 2016 [15]	↑ Fragmentation index	315	Actigraphy → autopsy	Arteriolosclerosis (OR 1.27, 95% CI: 1.11–1.45); subcortical infarcts (OR 1.31, 95% CI: 1.01–1.71)	Subcortical WM	Autopsy-confirmed neuropathology
ARCHITECTURE (PSG)						
Baril et al., 2021 [23]	↓ Slow-wave sleep	492	PSG	↓ Total brain (β = −4.21 cm^3^/1% SWS); ↓ frontal cortex volume	Global, frontal	FHS Offspring; 17-year prospective
Carvalho et al., 2023 [24]	↑ REM latency and ↑ Light sleep % (N2)	842	WatchPAT (home sleep test)	↓ AD-signature cortical thickness; ↑ WMH volume	Temporal, parietal, WM	Diverse cohort (HABS-HD); REM-specific effects and Inverse: ↑ deep sleep % = ↑ thickness (protective)
OSA						
Li et al., 2025 [25]	↑ AHI severity	40	PSG + fMRI	↓ fALFF/ReHo (cerebellum/frontal); ↑ occipital activity	Cerebellum, frontal, occipital	Mendelian randomization supports causality
Macey et al., 2010 [24]	Moderate-severe OSA	67	PSG (AHI 52.5 ± 5.3/h)	↓ GMC in the frontal, cingulate, thalamus, and hippocampus	Multiple cortical/subcortical	No GMV differences despite GMC changes
Khoroushi et al., 2024 [26]	OSA severity	40	PSG (AHI-based)	↑ White matter changes (ARWMC score, *p* < 0.001)	Global white matter	Independent of vascular risk factors
Carvalho et al., 2023 [24]	REM-specific AHI	842	WatchPAT	↑ WMH volume	Subcortical WM	REM apnea shows distinct WM vulnerability
Xiong et al., 2017 [12]	CPAP non-responders	29	PSG + DTI (≥30 d adherent)	Persistent ↑ MD in 17.8% WM tracts despite treatment	Widespread WM	30% incomplete structural recovery

AD, Alzheimer’s disease; AHI, apnea–hypopnea index; ARWMC, age-related white matter change; CPAP, continuous positive airway pressure; DTI, diffusion tensor imaging; FA, fractional anisotropy; fALFF, fractional amplitude low-frequency fluctuation; fMRI, functional MRI; FHS, Framingham Heart Study; GM, gray matter; GMC, gray matter concentration; GMV, gray matter volume; HABS-HD, Harvard Aging Brain Study-Health Disparities; MD, mean diffusivity; N2, stage 2 non-REM sleep; OSA, obstructive sleep apnea; PSG, polysomnography; ReHo, regional homogeneity; REM, rapid eye movement; SF, sleep fragmentation; SWS, slow-wave sleep; WM, white matter; WMH, white matter hyperintensity. N/A: not applicable; ↑: increase; ↓: decrease; → results in.

**Table 5:** Mechanistic studies: glymphatic function and neuroinflammation.

Mechanism	Ref.	N	Method	Key Finding	Sleep Association	Clinical Implication
Glymphatic Clearance						
DTI-ALPS index	[65]	31 AD	DTI perivascular diffusivity	↓ ALPS in AD correlates with MMSE	N/A	Validates the DTI-ALPS method
DTI-ALPS index	[66]	OSA cohort	PSG + DTI-ALPS	↓ ALPS correlates with AHI severity	OSA impairs the glymphatic	Dose–response relationship
DTI-ALPS index	[67]	72 (36 insomnia)	PSG + DTI-ALPS	↓ ALPS in insomnia correlates with TST	Total sleep time is critical	Affects memory
DTI-ALPS index	[67]	Mild TBI cohort	PSQI, clinical symptoms	ALPS increases from acute → chronic as sleep improves	Sleep recovery → glymphatic recovery	Demonstrates reversibility
DTI-ALPS index	[68]	AD with/without sleep disorders	Sleep disorder diagnosis	Sleep disorders exacerbate glymphatic impairment in AD	Combined AD + sleep disorder → worse ALPS	Synergistic negative effects
CSF clearance	[13]	Healthy adults	Randomized crossover	Sleep ↓ CSF Aβ and tau vs. wakefulness	Sleep enhances clearance	Direct mechanistic evidence
Neuro-inflammation						
CRP, WBC, PLT, G/L ratio	[5]	27,500	Composite sleep score + biomarkers	Inflammation mediates 7–10% of sleep-BAG	Poor sleep → inflammation → BAG	90% mechanistically unexplained
Vascular						
WMH burden	Carvalho et al. (2023) [24]	842	WatchPAT + MRI	↑ REM-AHI → ↑ WMH volume	REM-specific apnea effect	Small vessel disease pathway
Arteriolosclerosis	Lim et al. (2016) [15]	315	Actigraphy → autopsy	↑ Fragmentation → arteriolosclerosis (OR 1.27)	Chronic fragmentation	Neuropathological confirmation
WM microstructure	Ning et al. (2023) [22]	26,354	Self-report + DTI	Sleep behaviors → WMH and microstructural injury	Cross-sectional and longitudinal validation	Multiple sleep parameters contribute
Endothelial dysfunction	Schammel et al. (2022) [69]	Review/meta	OSA + WMH correlation	OSA is associated with WMH burden	Unclear if causation or correlation	Requires intervention trials

The DTI-ALPS index measures glymphatic function via perivascular space diffusivity; lower values indicate impaired clearance. Abbreviations: Aβ, amyloid-beta; AHI, apnea–hypopnea index; ALPS, analysis along perivascular space; BAG, brain age gap; CRP, C-reactive protein; DTI, diffusion tensor imaging; G/L, granulocyte-to-lymphocyte; MMSE, Mini-Mental State Examination; OSA, obstructive sleep apnea; PLT, platelet count; PSQI, Pittsburgh Sleep Quality Index; PSG, polysomnography; REM, rapid eye movement; TST, total sleep time; WBC, white blood cell count; WMH, white matter hyperintensity. N/A: not applicable; ↑: increase; ↓: decrease; → results in.

**Table 6:** Population diversity and generalizability assessment.

Study Cohort	Primary Studies Using Cohort	N	Ethnicity Distribution	Geographic Location	Age Range	SES Indicators	Generalizability Limitations
UK Biobank	Miao et al. [5], Tai et al. [19], Wang et al. (2024) [21], multiple others	27,500–479,420	~95% White European, ~5% other	United Kingdom	40–69 y	Higher education (>50% degree holders) is associated with better health than the general population.	Selection bias, limited ethnic diversity, and healthy volunteer bias
CARDIA	Cavaillès et al. (2024) [6]	619	48% Black, 52% White	USA (4 cities)	40 → 55 y (15 y follow-up)	Diverse SES; community-based	Better diversity than UK Biobank; moderate sample size
HABS-HD (Dormir)	Carvalho et al. (2023) [24]	842	Diverse (Hispanic, African American, White)	USA (California)	55–80 y	Diverse SES	Best diversity among major cohorts
SOL-INCA	Gonzalez et al. (2024) [20]	1005–2334	100% Hispanic/Latino (diverse backgrounds)	USA (4 cities)	35–85 y	Diverse SES; immigrant populations	Excellent Hispanic/Latino representation; limited to one ethnic group
Lifebrain	Fjell et al. (2023) [7]	3893 (8153 MRIs)	Predominantly European	Multisite European	Adult range	Generally higher SES	European-centric; selection bias

Abbreviations: CARDIA, Coronary Artery Risk Development in Young Adults; HABS-HD, Harvard Aging Brain Study-Health Disparities; MRI, magnetic resonance imaging; SES, socioeconomic status; SOL-INCA, Study of Latinos-Investigation of Neurocognitive Aging; y, years.

## Data Availability

No new data were created or analyzed in this study.

## Supplementary Materials

The following supporting information can be downloaded at: https://www.mdpi.com/article/10.3390/brainsci15121325/s1, Table S1: Methodological considerations and study quality.

## Abbreviations

The following abbreviations are used in this manuscript:

Aβ	Amyloid beta
ALFF	Amplitude of Low-Frequency Fluctuations
AD	Alzheimer’s disease
ADNI	Alzheimer’s Disease Neuroimaging Initiative
AHI	Apnea–hypopnea index
ALPS	Analysis along the perivascular space
ARIC	Atherosclerosis Risk in Communities
ARWMC	Age-related white matter change
BAG	Brain age gap
CARDIA	Coronary Artery Risk Development in Young Adults
CBF	Cerebral blood flow
CNN	Convolutional neural network
CPAP	Continuous positive airway pressure
CRP	C-reactive protein
CSF	Cerebrospinal fluid
DMN	Default mode network
DTI	Diffusion tensor imaging
FA	Fractional anisotropy
fALFF	Fractional amplitude of low-frequency fluctuation
FHS	Framingham Heart Study
fMRI	Functional MRI
G/L	Granulocyte-to-lymphocyte
GM	Gray matter
GMC	Gray matter concentration
GMV	Gray matter volume
HABS-HD	Harvard Aging Brain Study-Health Disparities
MAE	Mean absolute error
MD	Mean diffusivity
ML	Machine learning
MMSE	Mini-Mental State Examination
MRI	Magnetic Resonance Imaging
N2	Stage 2 non-REM sleep
OSA	Obstructive sleep apnea
PLT	Platelet
PSG	Polysomnography
PSQI	Pittsburgh Sleep Quality Index
RCT	Randomized controlled trial
ReHO	Regional Homogeneity
REM	Rapid eye movement
RST	Randomized controlled trial
SF	Sleep fragmentation
SPM	Statistical Parametric Mapping
SWS	Slow-wave sleep
TBSS	Tract-based spatial statistics
TSD	Total sleep deprivation
TST	Total sleep time
WASO	Wake after sleep onset
WBC	white blood cell
WM	White matter
WMH	White matter hyperintensity
