# Supplementary material for: Brain Age Acceleration on MRI Due to Poor Sleep: Associations, Mechanisms, and Clinical Implications
Source: Brain Sci. 2025 Dec 12;15(12):1325. doi: 10.3390/brainsci15121325 (PMC12730621; doi:10.3390/brainsci15121325)
Supplement: Supplementary file 1 [file brainsci-15-01325-s001.zip › brainsci-4022932-supplementary.pdf]

Brain Age Acceleration on MRI from Poor Sleep: Associations, Mechanisms, and Clinical Implications

Eman A. Torih [1,2], Mohammad H. Hussein [3], Abdulrahman Omar A. Alali [4], Asseel Farhan K. Alanazi [4], Nasser Rakan Almjlad [4], Turki Helal D. Alanazi [4], Rawaf Awadh T. Alanazi [4,] and Manal S. Fawzy [5,*]

Supplementary Table S1. Methodological considerations and study quality.

Methodological Domain	Key Issues	Impact on Interpretation	Recommendations
**Brain Age Algorithms**	Heterogeneous ML architecture; variable training datasets; inconsistent preprocessing	Algorithm-dependent associations; limits cross-study comparability	Standardize methods; validate against clinical outcomes; multi-algorithm sensitivity analyses
**Sleep Assessment**	90% of studies use self-report; subjective-objective discordance is well-established	Measurement error, recall bias, and misclassification	Prioritize PSG/actigraphy; validate self-report against objective measures
**Study Design**	85% cross-sectional or retrospective; limited longitudinal data	Cannot establish causality; reverse causation is possible	Prospective cohorts; intervention RCTs with serial MRI
**Population Diversity**	80% of UK Biobank (White European, educated, healthy)	Limited generalizability; selection bias	Replicate in diverse ethnic, socioeconomic, and geographic populations
**Effect Sizes**	BAG: 1-3 years (modest vs ~50-year aging span); sleep explains ≤21% variance	Clinical significance uncertain; most variance unexplained	Define clinically meaningful thresholds; identify additional mechanisms
**Mechanistic Evidence**	90% of the sleep-BAG association is mechanistically unexplained	Limits therapeutic targeting	Multimodal studies: MRI + PET + CSF + plasma biomarkers
**Intervention Evidence**	Zero RCTs of CBT-I/sleep extension on brain age; CPAP data limited	Cannot recommend sleep optimization for neuroprotection	Urgent need for intervention trials with MRI outcomes

Percentages are approximate estimates from a systematic review of cited literature.
